# Ex-vivo forces associated with intrauterine device placement and perforation: a biomechanical evaluation of hysterectomy specimens

**DOI:** 10.1186/s12905-021-01285-6

**Published:** 2021-04-07

**Authors:** Jane Duncan, Kathryn Fay, Jessica Sanders, Benjamin Cappiello, Jane Saviers-Steiger, David K. Turok

**Affiliations:** 1 Department of Obstetrics and Gynecology, University of Utah School of Medicine, 30 N 1900 E, Salt Lake City, UT 84132 USA; 2 Bioceptive, Inc, 1441 Canal St, New Orleans, LA 70112 USA

**Keywords:** Contraception, Intrauterine devices, Uterine perforation, Mechanical stress

## Abstract

**Background:**

This biomechanical analysis of hysterectomy specimens assesses the forces associated with intrauterine device placement. These include compressive forces required to cause uterine perforation with two commonly available commercial intrauterine device placement instruments and a metal uterine sound.

**Methods:**

We obtained hysterectomy specimens at a single academic center. All specimens resulted from excision for benign conditions in premenopausal women by any operative method. Within one hour of excision, we stabilized uterine specimens in an apparatus specifically designed for this analysis. A single, experienced clinician performed all experimental maneuvers and measured forces with a Wagner FDIX-25 force gauge. The investigator applied traction on a tenaculum to approximate force used during an intrauterine device placement. The maximum compressive force to the uterine fundus was determined by using manufacturers’ placement instruments for two commercially available products and a metal sound.

**Results:**

Sixteen individuals provided hysterectomy specimens. No complete perforations occurred while using loaded intrauterine devices; in a single observation the LNG IUS entered the myometrium. The plastic intrauterine device placement rod bowed in all attempts and did not perforate the uterine serosa at the fundus. A metal uterine sound created a complete perforation in all specimens (p < .001). The lowest mean maximum force generated occurred with the levonorgestrel intrauterine system placement instrument 12.3 N (SD ± 3.8 N), followed by the copper T380A intrauterine device placement instrument 14.1 N (SD ± 4.0 N), and highest for the metal sound 17.9 N (SD ± 7.6 N) (p < 0.01).

**Conclusions:**

In this ex-vivo model, metal uterine sounds caused complete perforation and intrauterine device placement instruments did not.

This study received Institutional Review Board (IRB0059096) approval.

## Background

In the United States, more individuals use intrauterine devices (IUDs) than ever before; patients frequently desire these methods for their excellent contraceptive efficacy, low user maintenance, and non-contraceptive benefits [1, 2]. Complications are rare, but primary among them are uterine perforations, occurring in an estimated 1 out of every 1000 placements [3, 4]. Perforations most likely occur at the time of placement, related to placement instruments and the force generated by such instruments [5, 6]. While many studies have examined IUD perforation rates, few studies have evaluated what aspect of the placement procedure causes perforation [7]. Manufacturer instructions and educational texts advise pelvic examinations, use of a uterine sound to measure cavity depth, and, if needed, an ultrasound as part of standard IUD placement practice [8–10]. However, scant data support these practices in achieving safe and correct placement. The use of the rigid metal sound is suspected to be the most frequent mechanism of perforation, as IUD placement instruments use plastic tubing that is flexible and is suspected to bow when obstructed [6, 11].

This study aims to assess the forces involved in IUD placement by examining the force generated by the placement instruments of two common IUDs and that of a metal sound. We hypothesize that the sound is capable of generating significant force and may present the major risk of perforation. We used a biomechanical analysis to compare force generation and potential for perforation with IUD placement alone versus metal sounds in hysterectomy specimens. The objectives of the study are two-fold: (1) to describe the maximum applied force generated by (a) the plastic IUD placement instruments and (b) a metal sound and (2) to determine incidence of uterine perforation when using maximum force generated by the manufacturers’ plastic IUD placement instruments compared to a metal sound.

## Methods

The research team devised an ex-vivo protocol utilizing hysterectomy specimens stabilized in a pelvic model apparatus to test the associated forces of IUD placement instruments. Trained study staff at a single academic institution approached patients scheduled for hysterectomy to obtain written consent for uterus collection. Inclusion criteria were premenopausal status and surgical completion of a total hysterectomy. Exclusion criteria included postmenopausal status, anatomic abnormalities, supra-cervical specimens, preserved specimens, conditions that would be a contraindication to IUD use per Centers for Disease Control and Prevention Medical Eligibility Criteria (categories 3 and 4) and inability to give informed consent [12]. In this pilot study, we set a goal sample size based on prior research in which five to twenty observations of each type of IUD were tested [6]. For this study, we planned to maximize enrollment over two years aiming to obtain three measurements for each of the three testing methods from at least ten participants. This study received Institutional Review Board approval (IRB0059096).

A single clinician-researcher with extensive IUD placement experience force-tested all collected specimens within one hour of excision. The instruments tested included a levonorgestrel (LNG) 52 mg intrauterine system (IUS) placement instrument (Mirena^®^, Bayer HealthCare Pharmaceuticals), a copper T380A IUD (ParaGard^®^, Teva Women’s Health, Inc.) placement instrument, and a metal uterine sound. We used a Wagner FDIX-25 force gauge for all measurements. Regarding nomenclature, we use the term IUD to refer to intrauterine contraception generically and specifically to the copper IUD. We distinguish hormonal delivery within the uterus and its unique mechanism of action by using the term IUS.

**Fig. 1:**
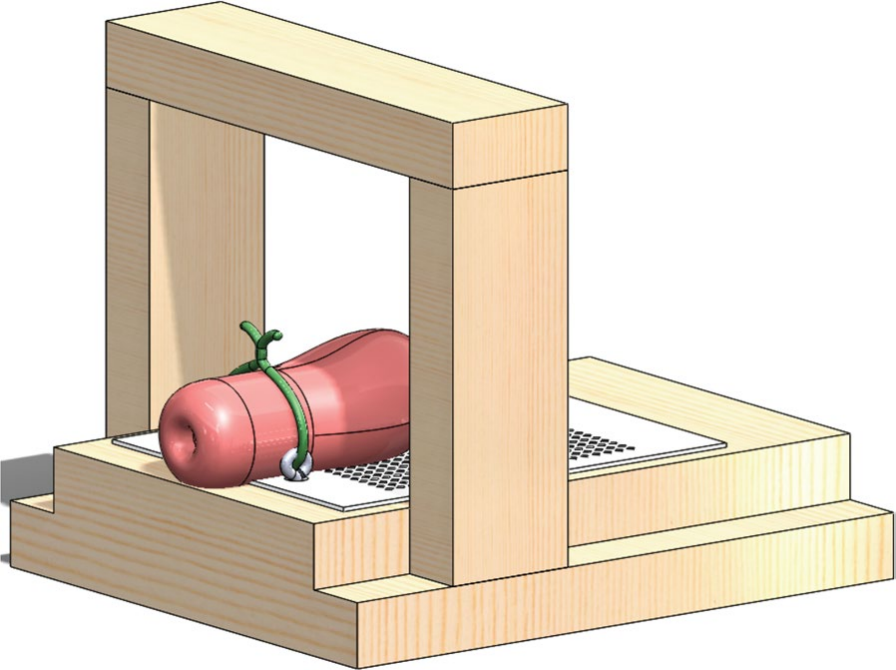
Uterus stabilization device

A member of the investigative team attached the hysterectomy specimens to the pelvic apparatus via a clamp on the cervix for stabilization (Fig. 1). This apparatus was designed in conjunction with a biomedical engineer and a family planning clinician to replicate in-vivo conditions. The clinical investigator connected each instrument to the force gauge and placed it at the external os. The investigator applied traction on a tenaculum to approximate force used during an intrauterine device placement. The researcher then advanced the instrument unit through the cervix and continued to advance until the instrument perforated the uterine corpus or, with maximum force, bowed and was unable to advance further. We recorded the maximum force value and then removed the instrument. The clinician tested each instrument three times before moving on to the next instrument, following the same protocol. The order of testing was LNG IUS, TCu380A IUD followed by metal sound for all specimens. Given our hypothesis that the uterine sound is the primary cause of IUD perforation at the time of placement, we completed this trial last. Perforation was gauged as visualization of the instrument external to the serosa. We collected data on the variables of IUD placement instruments, specimen size, patient parity, patient age, as well as patient diagnoses. We established the primary outcome as perforation force for the test instruments, generating mean values and standard deviations for each measured outcome. We compared perforation risk between the groups using Chi-square tests for categorical variables and compared maximum force using Wilcoxon rank sum tests to assess group differences in continuous force variables. All analyses were performed with STATA SE version 14.0 statistical software program (College Station, TX, USA).

## Results

Sixteen individuals met inclusion criteria and consented to use of their total hysterectomy specimens for the purpose of this study between July 2013 and June 2015. Age, parity, uterine cavity depth findings, and preoperative diagnoses are shown in Table 1.

**Table 1:** Demographic information of participants

Characteristic	Mean	Range
Age (years, n = 16)	40.3	27–51
Parity (n = 16)	2.3	0–6
Uterine cavity depth (cm, n = 13)	8.4	6–10.5

Preoperative diagnosis: abnormal uterine bleeding (n = 6), pelvic pain (n = 6) pelvic organ prolapse (n = 4), suspected ovarian malignancy (n = 3) and endometriosis (n = 1). Participants could have more than one diagnosis

The number of observations per instrument varied as the available IUD placement instruments were used for three attempts unless damage to the device occurred (from 40 separate observations for the copper IUD to 51 for the LNG IUS). For the metal sound, the instrument perforated the uterus in every scenario. Thus, the maximum measured force was the force recorded immediately prior to perforation. For the IUD placement trials, no complete perforations occurred. Thus, the maximum measured force occurred immediately prior to bowing of the plastic IUD placement instrument. In one observation, the LNG IUS entered the myometrium and was visualized tenting the uterine serosa but never passed through the serosa. Perforation was significantly more likely to happen with the metal sound (N = 47/47) than placement instruments (TCu380A IUD N = 0/40, LNG IUS N = 0/51, p < 0.001). When including the LNG partial perforation in statistical analysis, the risk based on instrument choice remains (p < 0.001).

**Fig. 2:**
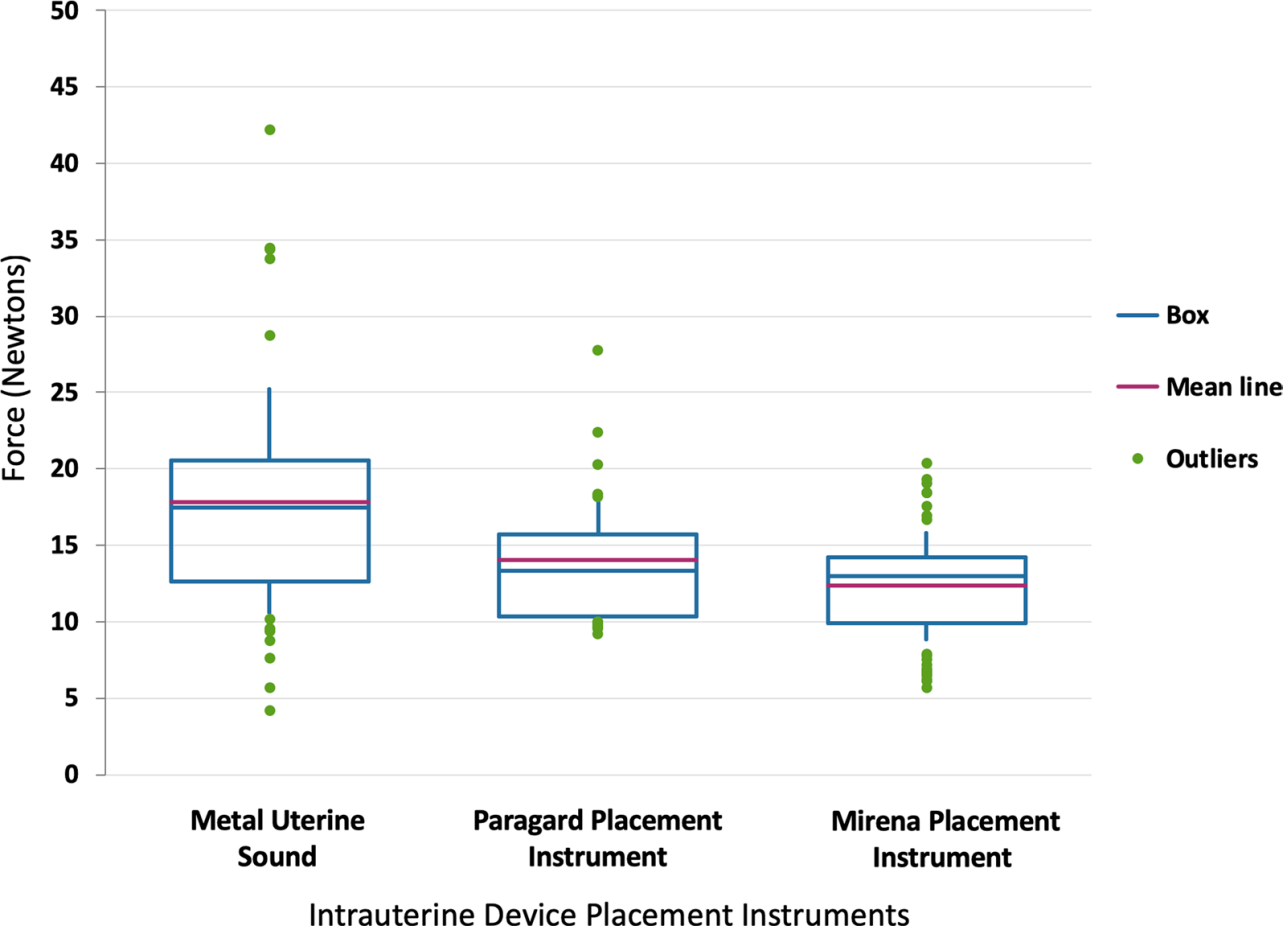
Box plot of max force generated with intent to perforate

Figure 2 reports the mean maximum forces generated for the instruments tested ranging from 12.3 Newtons (N, standard deviation 3.8 N) for the LNG IUS to the significantly greater 17.9 N (standard deviation 7.6 N, minimum 4.2 N, maximum 42.2 N) for the metal sound. The maximum force generated in all trials was with the metal sound of 42.2 N, 200 percent the maximum force generated with the LNG IUS (20.4 N) and 150 percent that of the CuT380A IUD (27.8 N). In three attempts with each of the IUD placement instruments (six total), the maximum force generated exceeded the mean maximum force generated by the metal sound but did not result in perforation.

## Discussion

In this ex-vivo model, metal uterine sounds caused complete perforations and IUD placement instruments did not. While the mean forces delivered with the metal sound exceed the mean of the IUD placement instruments, individual IUD attempts generated maximum forces greater than the mean force of perforation for the metal sound, but without perforation. The plastic sheath of contemporary IUD placement instruments bowed with excessive force and this may be a mechanism that preserves myometrial integrity that does not occur with a metal sound. In addition, a metal sound has greater density and a smaller surface area to distribute the force relative to the IUD placement instruments. Based on these findings, extrauterine IUD placement may be caused by IUDs passing through a defect created first by the uterine sound.

In 1987, Goldstuck published two studies using similar methodology assessing IUDs not currently available in the United States and a standard metal sound. He obtained data on the force required to place IUDs in-vivo and conducted ex-vivo testing on hysterectomy specimens as we did. Testing IUD placement instruments attempting to perforate ex-vivo specimens, he caused uterine perforation only with the rigid Lippes loop, finding that the Copper 250, Gravigard, Nova T, Lippes loop and Saf-T-Coil 25SX devices bowed before causing perforation [6, 11]. However, the forces generated in the current study with IUD placement instruments exceeded those reported by Goldstuck (5.75 N to 9.2 N) [5]. Similar to our findings, Goldstuck caused perforation with a metal sound with 100% of attempts when directed toward the fundus or cornua, generating a mean force of 20.7 N, approximating the mean value we obtained. Collectively, these prior data and the data presented from our study provide evidence that the metal sound increases perforation risk with IUD placement.

It is sensible to consider that the potential for perforation is greater with rigid, rather than hollow, insertion instruments. The implications of these finding support the continued study and consideration of simplified IUD placement techniques that avoid use of the uterine sound. While simplified techniques have been proposed with evidence of success, these have been performed by family planning experts, bringing into question its generalizability; the data from this study suggest that discontinuing the use of a metal uterine sound is even more important for inexperienced clinicians who are less familiar with reasonable force and tactile feedback in this setting [13]. In addition to potentially decreasing perforation risk, this approach could decrease pain [13]. Use of a uterine sound assures that the sound, and thus the IUD placement instrument, can be passed to the uterine fundus, evaluating both cervical stenosis and patient tolerance. Clinicians may use a variety of techniques to accomplish the same objective, including ultrasound guidance, local anesthesia, rapid technique, and “verbicaine” (a medical provider using language to decrease a patient’s anxiety and discomfort). Practitioners encountering difficulty passing the IUD through the cervical os may be more likely to succeed with use of a tapered dilator, an “os-finder,” rather than a metal sound [14]. The most compelling reason for using a uterine sound is to determine the size of the uterus. The mean uterine cavity length in a study of both nulligravida and parous patients was found to be greater than 3.6 cm and thus candidates for the 3.2 cm LNG-IUS or 3.6 cm copper IUD [15]. Uterine size is an uncommon contraindication to IUD use and can be ascertained by bimanual examination, or, if needed, ultrasound. Medical judgement for use in these rare circumstances is advised; regardless, a uterine sound would be immaterial in these decisions. The use of a single experienced clinician limits external validity and generalizability, but provides internal validity. Conducting ex-vivo testing within one hour of excision augments model fidelity, but does not approximate in-vivo IUD use.

This study reports a simulation clearly distinct from actual clinical care. This approach inherently drives certain limitations. We did not obtain force data generated with actual clinical care and we used a small number of specimens, though the limited sample size generated statistically significance results. In theory it is possible that the prior trials of IUD placement devices weakened the myometrium. Even if this were the case, we feel the conclusions drawn are still valid given the statistically significance difference in generated force. A possible limitation in generalizability is the exclusion of specimens with anatomical anomalies and the absence of specimens from nulligravid and parous women with a history of one or more Caesarean deliveries. Previous studies suggest that women less than 6 months postpartum are at risk of higher incidence of perforation with IUD placement; we did not evaluate this trend in the study [16]. IUD placement is more difficult in nulligravid women with tight cervices, and in parous women with history of one or more Caesarean delivery [17]. The pelvic apparatus (Fig. 1), although designed to replicate in-vivo conditions, does not account for retroverted or retroflexed uterine positions. All perforations occurred at the uterine fundus. This limited study does not portend discontinued use of uterine sounds, but instead invites dialogue and further research to determine what steps are necessary for appropriate placement of an IUD and how such steps may depend on the parity or uterine position of the patient.

We compared only two IUD placement instruments which represent the current majority of commercial products being utilized; however, new IUDs that we did not test are now FDA approved for use in the United States (Liletta^®^ Medicines360, Skyla^®^ Bayer, and Kyleena^®^ Bayer) and more are being assessed in FDA phase 3 trials [18–24]. Likely, these data apply to those methods but lack of testing creates lack of assurance. The Liletta^®^ has a distinct placement instrument and the Skyla^®^ and Kyleena^®^ have a narrower inserter which, although unlikely to change the results, warrant additional study. Study data collection did not include a perception of conventional force used during IUD placement relative to the maximum forces reported here; instead, the clinician reported that the maximum force used with each attempt far exceeded what he would ever consider using in real practice. This observation concurs with data demonstrating that in-vivo IUD placement required a force of 1.5 N to 4.0 N [6]. This study design permits force testing to failure, a major study strength that could not occur with in-vivo testing. These data provide insight into the likely main cause of uterine perforation: the metal sound.

## Conclusions

This work builds upon and modernizes prior ex-vivo studies with IUD products relevant to clinicians today. Application of these data may decrease perforation rates and potentially improve the already excellent safety profile of IUDs. IUDs have shown remarkable increase in uptake by contraceptive users and this trajectory is likely to continue, often with placement by clinicians other than high-volume, family planning specialists, highlighting the need for evidence-based simplification and improved safety around IUD placement. This study demonstrates potential liability of contemporary use of a metal uterine sound. The widespread use of metal sounds for IUD placement should be reevaluated as we provide a plausible mechanism for uterine perforation with IUD placement.

## Data Availability

The datasets used and/or analyzed during the current study are available from the corresponding author on reasonable request.

## Abbreviations

IUD: Intrauterine device
LNG: Levonorgestrel
IUS: Intrauterine system

## References

[1] Hubacher D, Kavanaugh M (2018). Historical record-setting trends in IUD use in the United States. Contraception.

[2] World Health Organization. Reproductive Health and Research, K4Health. Family planning: a global handbook for providers: evidence-based guidance developed through worldwide collaboration. Geneva Baltimore: World Health Organization John Hopkins Bloomberg School of Public Health, Center for Communication programs, Knowledge for Health Project. 2011. xii, 372 pp.

[3] Heinemann K, Reed S, Moehner S, Minh TD (2015). Risk of uterine perforation with levonorgestrel-releasing and copper intrauterine devices in the European Active Surveillance Study on Intrauterine Devices. Contraception.

[4] O’Brien PA, Pillai S (2017). Uterine perforation by intrauterine devices: a 16-year review. J Fam Plann Reprod Health Care.

[5] Goldstuck ND (1987). “Bowing” forces with IUD inserters in vitro: relevance to difficult IUD insertions. Clin Reprod Fertil.

[6] Goldstuck ND (1987). Insertion forces with intrauterine devices: implications for uterine perforation. Eur J Obstet Gynecol Reprod Biol.

[7] Jatlaoui TC, Riley HEM, Curtis KM (2016). The safety of intrauterine devices among young women: a systematic review. Contraception.

[8] Teva Pharmaceuticals. Paragard Package Insert [Internet]. 2005 [cited 2020 Apr 16]. http://www.accessdata.fda.gov/drugsatfda_docs/label/2005/018680s060lbl.pdf.

[9] Bayer HealthCare Pharmaceuticals. Mirena Package Insert [Internet]. 2008 [cited 2020 Apr 16]. https://www.accessdata.fda.gov/drugsatfda_docs/label/2008/021225s019lbl.pdf.

[10] Hatcher R, Dean G, Schwarz E. Intrauterine devices (IUDs). In: Contraceptive technology. 21st ed. Managing Contraception, LLC; 2018. p. 157–81.

[11] Goldstuck ND, Wildemeersch D (2014). Role of uterine forces in intrauterine device embedment, perforation, and expulsion. Int J Womens Health.

[12] Curtis KM, Tepper NK, Jatlaoui TC, Berry-Bibee E, Horton LG, Zapata LB (2016). U.S. medical eligibility criteria for contraceptive use, 2016. MMWR Recomm Rep.

[13] Christenson K, Lerma K, Shaw KA, Blumenthal PD (2016). Assessment of a simplified insertion technique for intrauterine devices. Int J Gynaecol Obstet.

[14] Dermish A, Turok DK, Jacobson J, Murphy PA, Saltzman HM, Sanders JN (2016). Evaluation of an intervention designed to improve the management of difficult IUD insertions by advanced practice clinicians. Contraception.

[15] Canteiro R, Bahamondes MV, dos Santos FA, Espejo-Arce X, Marchi NM, Bahamondes L (2010). Length of the endometrial cavity as measured by uterine sounding and ultrasonography in women of different parities. Contraception.

[16] Caliskan E, Oztürk N, Dilbaz BO, Dilbaz S (2003). Analysis of risk factors associated with uterine perforation by intrauterine devices. Eur J Contracept Reprod Health Care Off J Eur Soc Contracept.

[17] Santos ARG, Bahamondes MV, Hidalgo MM, Atti A, Bahamondes L, Monteiro I (2013). Pain at insertion of the levonorgestrel-releasing intrauterine system in nulligravida and parous women with and without cesarean section. Contraception.

[18] Bayer HealthCare Pharmaceuticals Inc. Skyla Package Insert [Internet]. 2016 [cited 2020 Jun 11]. https://www.accessdata.fda.gov/drugsatfda_docs/label/2017/203159s007lbl.pdf.

[19] Bayer HealthCare Pharmaceuticals Inc. Kyleena Package Insert [Internet]. 2016 [cited 2020 Jun 11]. https://www.accessdata.fda.gov/drugsatfda_docs/label/2016/208224s000lbl.pdf.

[20] Turok DK, Nelson AL, Dart C, Schreiber CA, Peters K, Schreifels MJ (2020). Efficacy, safety, and tolerability of a new low-dose copper and nitinol intrauterine device: phase 2 data to 36 months. Obstet Gynecol.

[21] Sebela Pharmaceuticals Inc. A Phase 3, Prospective, Multi-Center, Single-Arm, Open-Label Study to Evaluate VeraCept^TM^, a Long-Acting Reversible Intrauterine Contraceptive for Contraceptive Efficacy, Safety, and Tolerability [Internet]. [cited 2020 Jun 11]. https://clinicaltrials.gov/ct2/show/NCT03633799.

[22] Sebela Pharmaceuticals Inc., Turok DK. Evaluation of the Effectiveness, Safety and Tolerability of LevoCept (Levonorgestrel-Releasing Intrauterine System) for Long-Acting Reversible Contraception [Internet]. [cited 2020 Jun 11]. https://clinicaltrials.gov/ct2/show/NCT02882191.

[23] Blithe, D, Hubacher, D. A Multi-center, Single-blind, Randomized Clinical Trial to Compare Two Copper IUDs: Mona Lisa NT Cu380 Mini and ParaGard [Internet]. [cited 2020 Jun 11]. https://clinicaltrials.gov/ct2/show/NCT03124160.

[24] Odyssea Pharma, SPRL, Belgium, Allergan USA, Inc. Liletta Package Insert [Internet]. 2018 [cited 2020 Jun 11]. https://www.accessdata.fda.gov/drugsatfda_docs/label/2018/206229s007lbl.pdf.

